# Genome-wide methylation and transcriptome of blood neutrophils reveal the roles of DNA methylation in affecting transcription of protein-coding genes and miRNAs in *E. coli*-infected mastitis cows

**DOI:** 10.1186/s12864-020-6526-z

**Published:** 2020-01-30

**Authors:** Zhihua Ju, Qiang Jiang, Jinpeng Wang, Xiuge Wang, Chunhong Yang, Yan Sun, Yaran Zhang, Changfa Wang, Yaping Gao, Xiaochao Wei, Minghai Hou, Jinming Huang

**Affiliations:** 1 Dairy Cattle Research Center, Shandong Academy of Agricultural Sciences, Jinan, Shandong 250131 People’s Republic of China; 2 Engineering Center of Animal Breeding and Reproduction, Jinan, Shandong 250100 People’s Republic of China

**Keywords:** Cows, *E. coli* mastitis, Neutrophils, DNA methylation, Expression pattern, miRNA, Alternative splicing, Immune responses

## Abstract

**Background:**

Neutrophils are the first effectors of inflammatory response triggered by mastitis infection, and are important defense cells against pathogenic *Escherichia coli* (*E. coli*). DNA methylation, as a critical epigenetic mechanism for regulating gene function, is involved in bovine mastitis.

**Results:**

In this study, we sequenced the blood neutrophils of healthy and *E. coli-*infected mastitic half-sib cows for the overall DNA methylation levels using transcriptome sequencing and reduced representation bisulfite sequencing. The methylation levels in the mastitis cows (MCs) were decreased compared with healthy cows (HCs). A total of 494 differentially methylated regions were identified, among which 61 were up-methylated and 433 were down-methylated (MCs vs. HCs). The expression levels of 1094 differentially expressed genes were up-regulated, and 245 genes were down-regulated. Twenty-nine genes were found in methylation and transcription data, among which seven genes’ promoter methylation levels were negatively correlated with expression levels, and 11 genes were differentially methylated in the exon regions. The bisulfite sequencing PCR and quantitative real-time PCR validation results demonstrated that the promoter methylation of *CITED2* and *SLC40A1* genes affected differential expression. The methylation of *LGR4* exon 5 regulated its own alternative splicing. The promoter methylation of bta-miR-15a has an indirect effect on the expression of its target gene *CD163*. The *CITED2*, *SLC40A1*, and *LGR4* genes can be used as candidates for *E. coli*-induced mastitis resistance.

**Conclusions:**

This study explored the roles of DNA methylation in affecting transcription of protein-coding genes and miRNAs in *E. coli*-induced mastitis, thereby helping explain the function of DNA methylation in the pathogenesis of mastitis and provided new target genes and epigenetic markers for mastitis resistance breeding in dairy cattle.

## Background

Mastitis, one of the most prevalent diseases in the dairy cattle industry, leads to great economic losses caused by reduced milk production, discarded milk, early culling, veterinary services, and labor costs [1]. Mastitis is the inflammation of the mammary gland caused by various pathogenic bacteria. *Escherichia coli* (*E. coli*), as one of the most common environmental pathogens, is the cause of acute clinical mastitis. The *E. coli*-infected mastitic cows presents serious systemic clinical symptoms; the disease may cause several deaths per year in the most severe cases [2].

The mastitis-causing pathogens induce inflammation responses in the mammary gland; therefore, the host produces an immune responses for protection. An effective immune response begins when the bacteria interact with cells within the mammary gland, including epithelial cells and leukocytes [3]. The first leukocytes to migrate into the mammary gland in large numbers are neutrophils. Neutrophils are essential to immune response, as they are the initial phagocytic cells to arrive at an infection site to begin phagocytosis [4]. Neutrophils, as part of the innate immune response, comprise the majority of white blood cells. They are present in the bloodstream until signaled to an infection site by the body’s chemical cues. Neutrophils act rapidly, arriving at the site of infection within an hour through a chemotaxis process [5]. Migration of neutrophils into the mammary gland provides the first line of defense against invading mastitis pathogens [6].

In response to the inflammation from bovine mastitis, the host secretes corresponding cytokines that cause changes in the regulation of gene expression [3]. DNA methylation plays a crucial role in regulating gene expression. DNA methylation, as an epigenetic modification mark, can change the gene activity but does not affect the DNA sequence. DNA methylation participates in many biological processes, including genomic imprinting and inflammatory disease [7]. DNA methylation is involved in the occurrence and development of bovine mastitis [8–10]. DNA methylation differences of peripheral blood lymphocytes have been observed between *Staphylococcus aureus*-infected mastitic cows and healthy cows [11].

Genome-wide DNA methylation level of human neutrophils infected by the *Anaplasma phagocytophilum* pathogen has been observed to significantly increase [12]. Moreover, genome-wide DNA methylation map of human neutrophils reveals widespread inter-individual epigenetic variations, and variations in DNA methylation pattern in different individuals alter disease susceptibility [13].

Understanding the epigenetic regulation differences in mastitis susceptibility is important. Therefore, we used reduced representation bisulfite sequencing (RRBS) and transcriptome sequencing (RNA-seq) to identify and compare genome-wide DNA methylation and transcription patterns of blood neutrophils from three healthy and three *E. coli*-infected mastitis half-sib cows. We also identified the key differentially methylated and differentially expressed genes involved in *E. coli* mastitis, and investigated the DNA methylation regulation mechanisms underlying the immune response in dairy cattle. The present study aimed to analyze the genome-wide DNA methylation patterns of blood neutrophils in healthy and *E. coli*-infected mastitis cows, and to identify new epigenetic markers for mastitis resistance breeding.

## Results

### Experimental samples identification and analysis

**Fig. 1:**
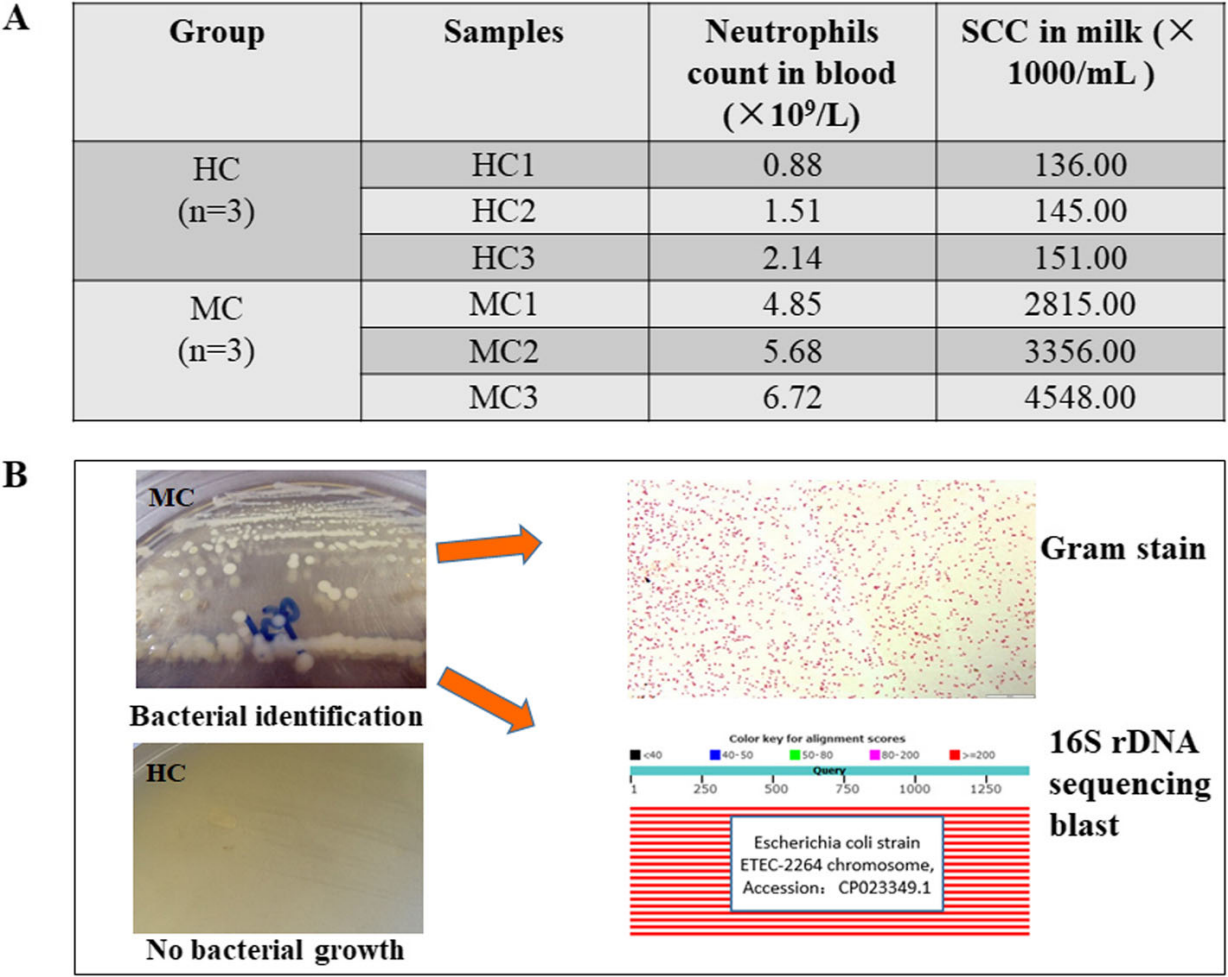
Basic information of the three healthy and three *E. coli*-infected mastitic cow samples. **a** The milk SCCs records and blood neutrophil counts results of six cow samples. **b** The bacterial identification results of six cow samples. Note: HC, Healthy cow; MC, Mastitic cow; HC1, Healthy cow 1; HC2, Healthy cow 2; HC3, Healthy cow 3; MC1, Mastitic cow 1; MC2, Mastitic cow 2; MC3, Mastitic cow 3; SCCs, Somatic cell counts. The definitionss in the following figures are the same as the first one

The half-sibling matched three healthy cows (HC groups), and three *E. coli* infected mastitic cows (MC groups) were selected from a total of 52 candidate samples based on milk SCCs, hematological analysis, and bacterial identification result. As shown in Fig. 1a, the average blood neutrophil count of MC group cows was 5.75 × 10^9^/L, which was significantly higher compared with the HC group cows that have an average blood neutrophil count of 1.51 × 10^9^/L (*P* < 0.05). The average SCC in the milk of MC group was 3.57 × 10^6^/mL, which was significantly higher than HC group 1.40 × 10^5^/mL (*P* < 0.05). In addition, we isolated and cultured bacteria from milk samples. In the healthy group, the presence of bacteria growth in the culture plate was not observed. In contrast, the culture plate of MC group samples displayed numerous single colonies and rod-shaped Gram-negative bacteria. The bacterial infection was identified as *E. coli* strain by 16S rDNA sequencing blast of bacterial genome DNA (Fig. 1b).

### Genome-wide DNA methylation patterns of bovine blood neutrophils

**Fig. 2:**
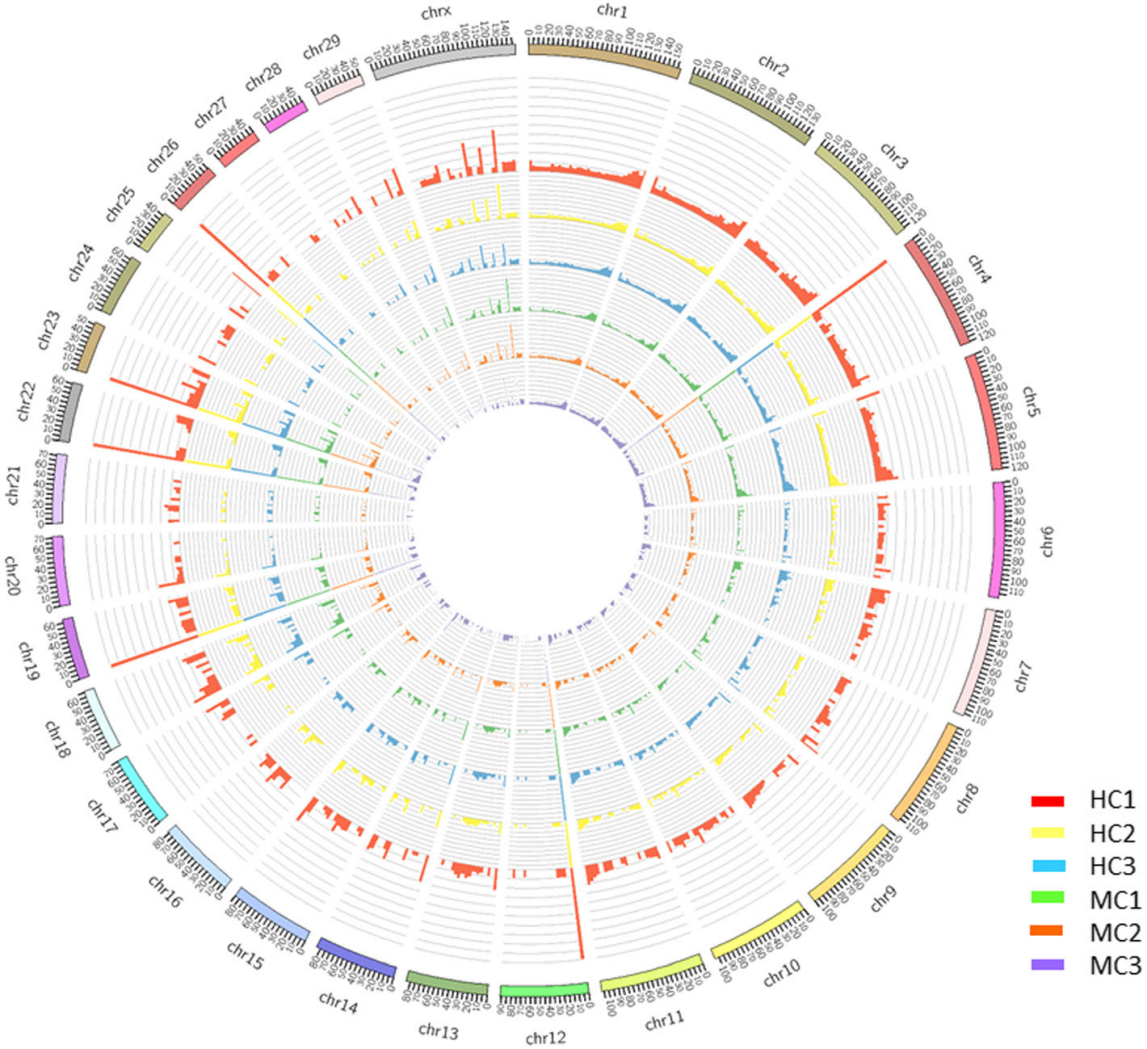
The overall DNA methylation maps of three HC samples (the outer three circles) and three MC samples (the inner three circles) across chromosomes. Note: The outer three circles showed the HC1, HC2, and HC3 samples, respectively. The inner three circles showed the MC1, MC2, and MC3 samples, respectively

We sequenced and analyzed genome-wide DNA methylation levels of bovine peripheral blood neutrophils of three healthy cows and three *E. coli*-infected mastitic cows using the RRBS method. The overall DNA methylation maps of three HC samples (the outer three circles) and three MC samples (the inner three circles) across chromosomes were drawn in Circos plot (Fig. 2). The figure showed that DNA methylation levels of *E. coli*-infected mastitic cows were lower than healthy cows.

**Fig. 3:**
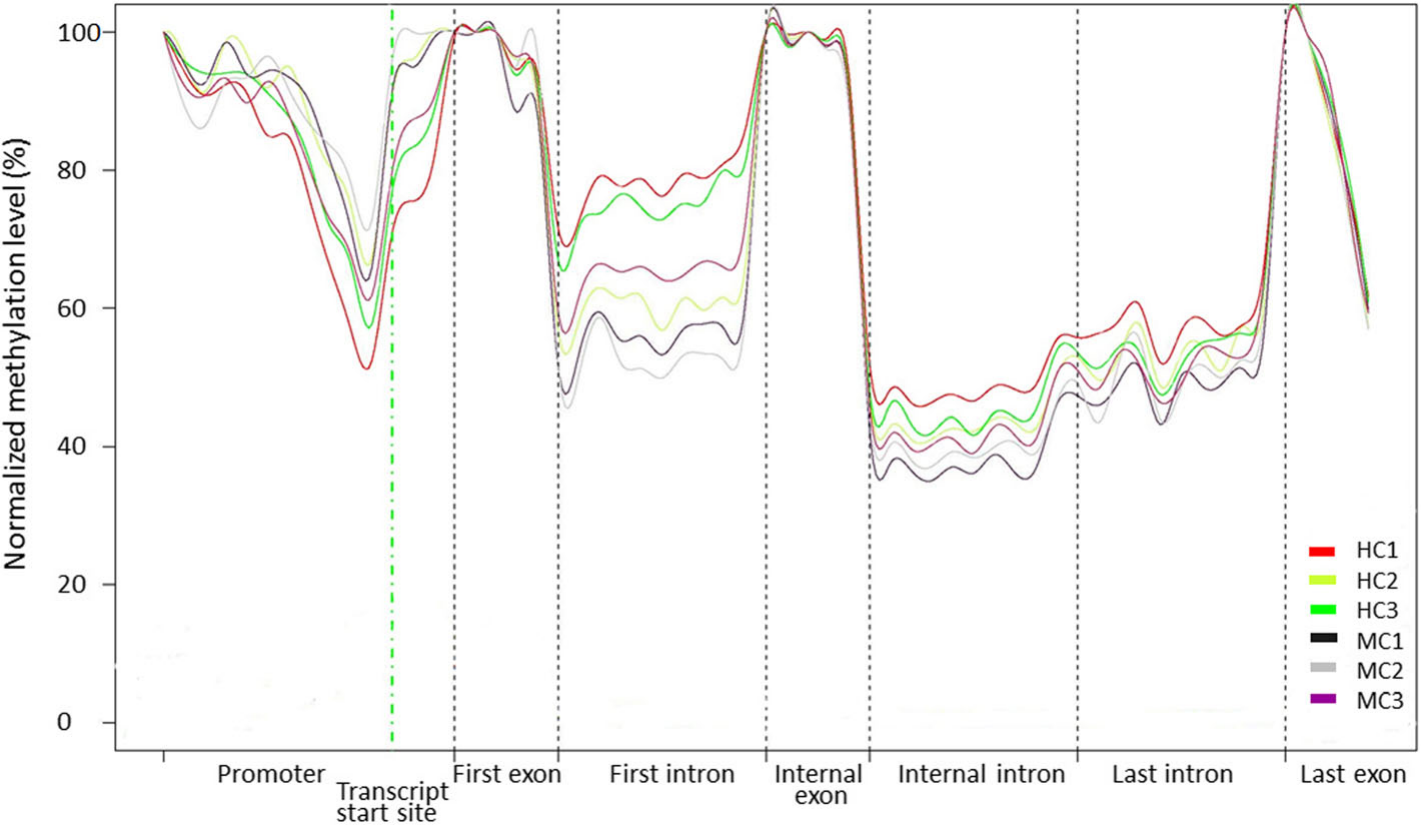
Distribution of DNA methylation level in different genome regions in six cow samples. The x axis indicates different gene elements, and the y axis indicates the normalized methylation levels of differentially methylated genes on specific gene element

In addition, the methylation levels in different genome regions, including promoter, first exon, first intron, internal exon, internal intron, last intron, and last exon, were analyzed to investigate methylation patterns in whole genome (Fig. 3). DNA methylation profiles within genomic regions revealed similar patterns across all sample types. The DNA methylation levels were higher in the promoter region, decreased dramatically around the transcript start site, and increased sharply toward the gene’s first exon. Furthermore, significantly higher methylation levels were observed in internal and last exons compared with internal and last introns.

### Differentially methylated regions between HC and MC groups

**Fig. 4:**
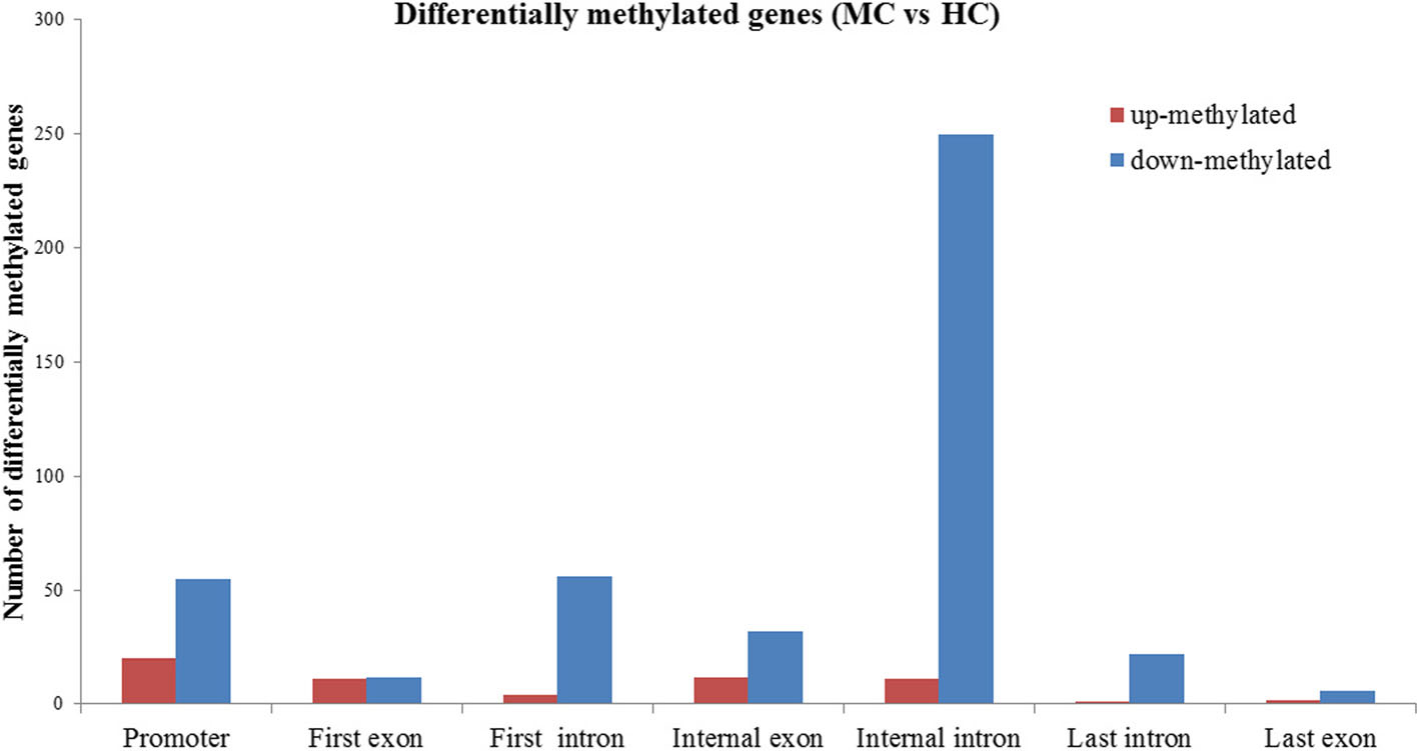
The number of differentially methylated genes on each gene element in healthy and mastitic cows

To identify differentially methylated regions, the methylated peaks between HC and MC groups were compared. The methylation ratio of total methylated genes and differentially methylated genes in healthy and mastitis cows were analyzed. The methylation levels of total methylated and differentially methylated genes in the mastitis cows were decreased compared with healthy cows. Consequently, a total of 494 differentially methylated regions, located at the 356 gene, were observed between HC and MC libraries (*P* < 0.05), of which 61 were up-methylated and 433 were down-methylated in mastitic cows compared with healthy cows (Additional file 1: Table S1). These results demonstrated that the number of differentially up-methylated genes was decreased compared with the number of differentially down-methylated genes (MC vs. HC). Of these DMRs, 75 regions were located with gene promoters, from which 20 genes were up-methylated and 55 genes were down-methylated. A total of 75 regions were located in the exons of genes, whereas 344 regions were located in the introns of genes (Fig. 4). To further understand the biological functions of differentially methylated genes, we performed GO and KEGG pathway analyses. Out of these genes, 52 differentially methylated genes were associated with immune, defense, and inflammation responses (Additional file 2: Figure S1A). To analyze the interaction between differentially methylated genes, we performed a protein interaction network analysis of 52 genes by STRING software, and determined that 14 genes were interacting with each other (Additional file 2: Figure S2A). Of these genes, a cluster of differentiation genes (*CD163*, *CD38*, and *CD86*), interleukin genes (*IL12A*, *IL1R1*, and *IL6R*), *LGR4*, and *RBPJ* genes were included.

In addition, by QTL location in AnimalQTL (http://www.animalgenome.org/cgi-bin/QTLdb/BT/search), we screened differential methylation genes that are located within cattle QTLs and identified a total of 69 differentially methylated genes harbored in the QTLs associated with clinical mastitis, somatic cell count and somatic cell score (Additional file 1: Table S2). For example, the *RBPJ* and HERC5 genes are located in the somatic cell score QTL #10439 region of chromosome 6. These genes were reportedly to be involved in immune, defense, and inflammation responses [14, 15]. The *RBPJ* and *HERC5* genes may play roles in the mastitis infection via DNA methylation’s influence. Therefore, they can be used as candidate genes for mastitis resistance studies.

We detected differentially methylated miRNAs between HC and MC libraries. Twenty-six differentially methylated miRNAs, including 8 up-methylated and 18 down-methylated miRNAs, were identified in the healthy and mastitic groups (Additional file 1: Table S3). Of these 26 miRNAs, bta-miR-146a and bta-miR-15a regulate immune and inflammation responses in bovine mastitis [16, 17].

### Differentially expressed genes between HC and MC groups

Using RNA-seq, we compared the transcriptomic landscapes of neutrophil from the healthy and *E. coli*-infected mastitic cows. According to the RNA-seq sequencing analysis, expression levels of 1094 DEGs were up-regulated, and 245 DEGs were down-regulated in mastitic cows compared with the healthy cows (Additional file 1: Table S4). Among these DEGs, 140 genes were involved in inflammatory, immune, and defense responses by GO analysis (Additional file 2: Figure S1B). In addition, a total of 415 differentially expressed genes were located in clinical mastitis, SCC, and SCS QTL regions. The 37 differentially expressed genes are located in the QTL regions related to mastitis, and involved in immune, inflammatory, and defense responses, among which 15 genes have protein interactions (Additional file 2: Figure S2B). These genes included chemokines (*CCL3* and *CCL4*), interleukin (*IL18* and *IL1A*), inflammasome (*NLRC4* and *NLRX1*), and tumor-necrosis factor superfamily (*TNF* and *TNFSF14*).

### Association analysis between methylation and transcriptome data

Considering the profound influence of DNA methylation on the regulation of gene expression, we examined whether differential methylation between healthy and mastitic cow groups might be the basis for gene expression differences. Therefore, the correlation analysis between DNA methylation and gene expression data in mastitic cows against healthy cows was performed. The methylation levels of promoter regions were negatively correlated with expression levels (Pearson’s *r* = − 0.12*, P* = 0.006 for MC group; and Pearson’s *r* = − 0.14, *P* = 0.003 for HC group) (Additional file 2: Figure S3).

**Fig. 5:**
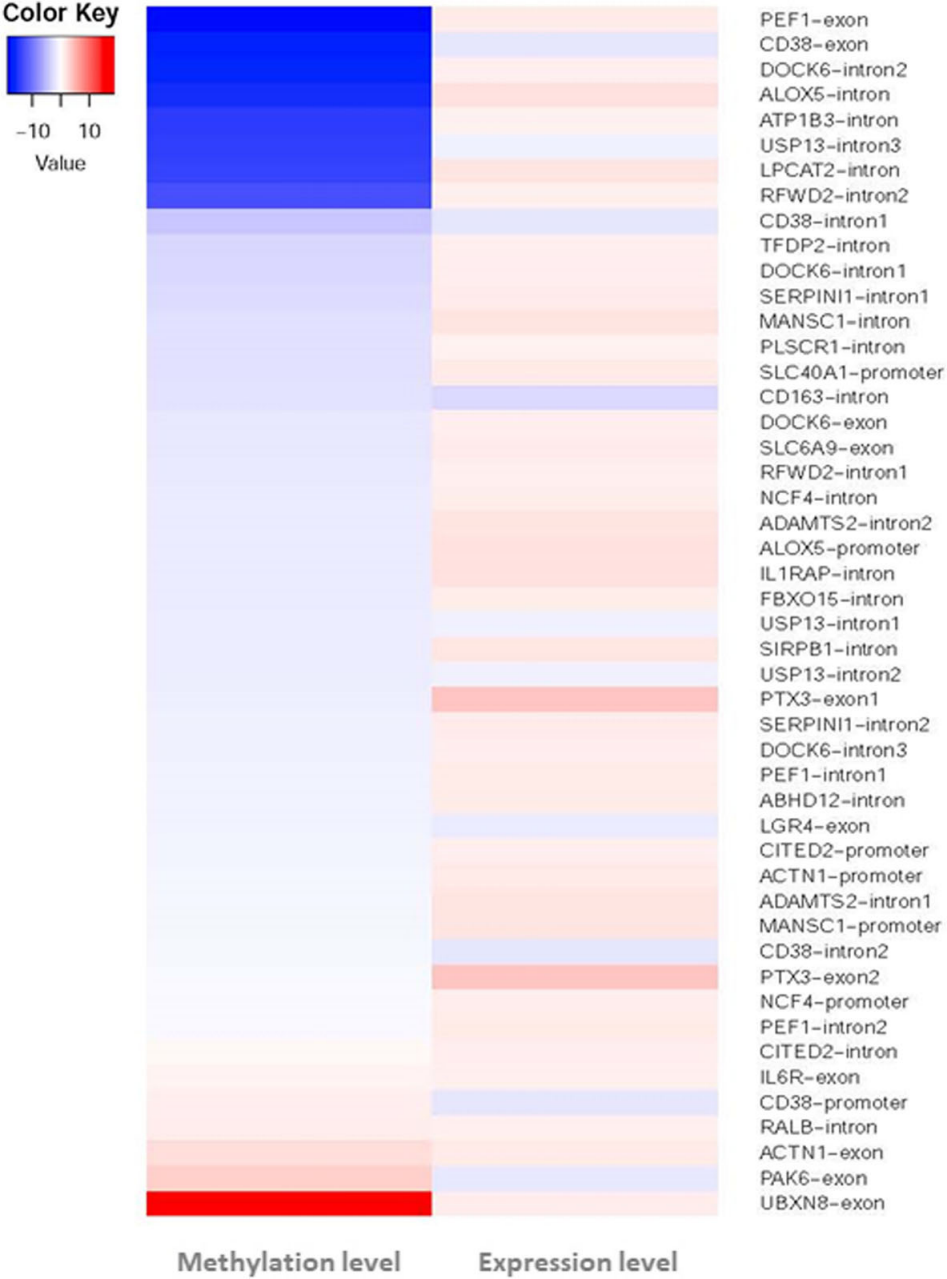
Heat map analysis of gene methylation and expression levels in cow neutrophils

Furthermore, the differentially expressed and -methylated genes were compared through integrated transcriptomic and methylomic analysis. A total of twenty-nine differential expressed and -methylated genes were found in both RNA-seq and RRBS sequencing (Fig. 5 and Additional file 1: Table S5). Of these genes, identified gene numbers in the promoter and exon differential methylated regions were 7 and 11, respectively. Seven differentially methylated and -expressed genes were associated with immune and inflammation responses in the RRBS and RNA-seq sequencing (Table 1), such as the *SLC40A1*, *CITED2*, and *LGR4* gene. The *CITED2* and *SLC40A1* genes were hypomethylated in the promoter regions and up-regulated mRNA expression. The *LGR4* gene was hypomethylated in the exon region and down-regulated mRNA expression. KEGG analysis of differentially expressed and -methylated genes showed that the twenty-nine genes could enrich seven signaling pathways (Additional file 2: Figure S4), such as the “leukocyte transendothelial migration” pathway. Transendothelial migration is necessary for the entry of neutrophils into the inflammation site.

**Table 1:** Differentially-methylated and -expressed genes associated with immune and inflammation responses in RRBS and RNA-seq sequencing

Chromosome	Ensembl Gene ID	Gene ID	Gene description
chr13	ENSBTAG00000001420	ABHD12	Abhydrolase domain containing 12
chr5	ENSBTAG00000019669	CD163	CD163 molecule
chr6	ENSBTAG00000013569	CD38	CD38 molecule
chr9	ENSBTAG00000011224	CITED2	Cbp/p300 interacting transactivator with Glu/Asp rich carboxy-terminal domain 2
chr3	ENSBTAG00000018474	IL6R	Interleukin 6 receptor
chr15	ENSBTAG00000002606	LGR4	Leucine rich repeat containing G protein-coupled receptor 4
chr5	ENSBTAG00000007531	NCF4	Neutrophil cytosolic factor 4
chr1	ENSBTAG00000009012	PTX3	Pentraxin 3
chr2	ENSBTAG00000032021	RALB	RAS like proto-oncogene B
chr2	ENSBTAG00000010498	SLC40A1	Solute carrier family 40 member 1

To investigate whether differentially methylated miRNAs account for gene differential expression, we predicted their potential target genes by TargetScan software. The targets of 26 miRNAs captured 84 differentially expressed genes in the HC and MC groups (Additional file 2: Figure S5). For example, bta-miR-15a was hypomethylated, whereas the expression level of its target gene *CD163* was down-regulated (MC vs. HC), thereby demonstrating that the methylation level of miRNA was positively correlated with its target gene’s expression level. The target genes expression might be influenced by the DNA methylation of miRNAs.

### The validation of candidate protein-coding genes and miRNAs

Methylation and expression level analysis of *CITED2* and *SLC40A1* genes.

**Fig. 6:**
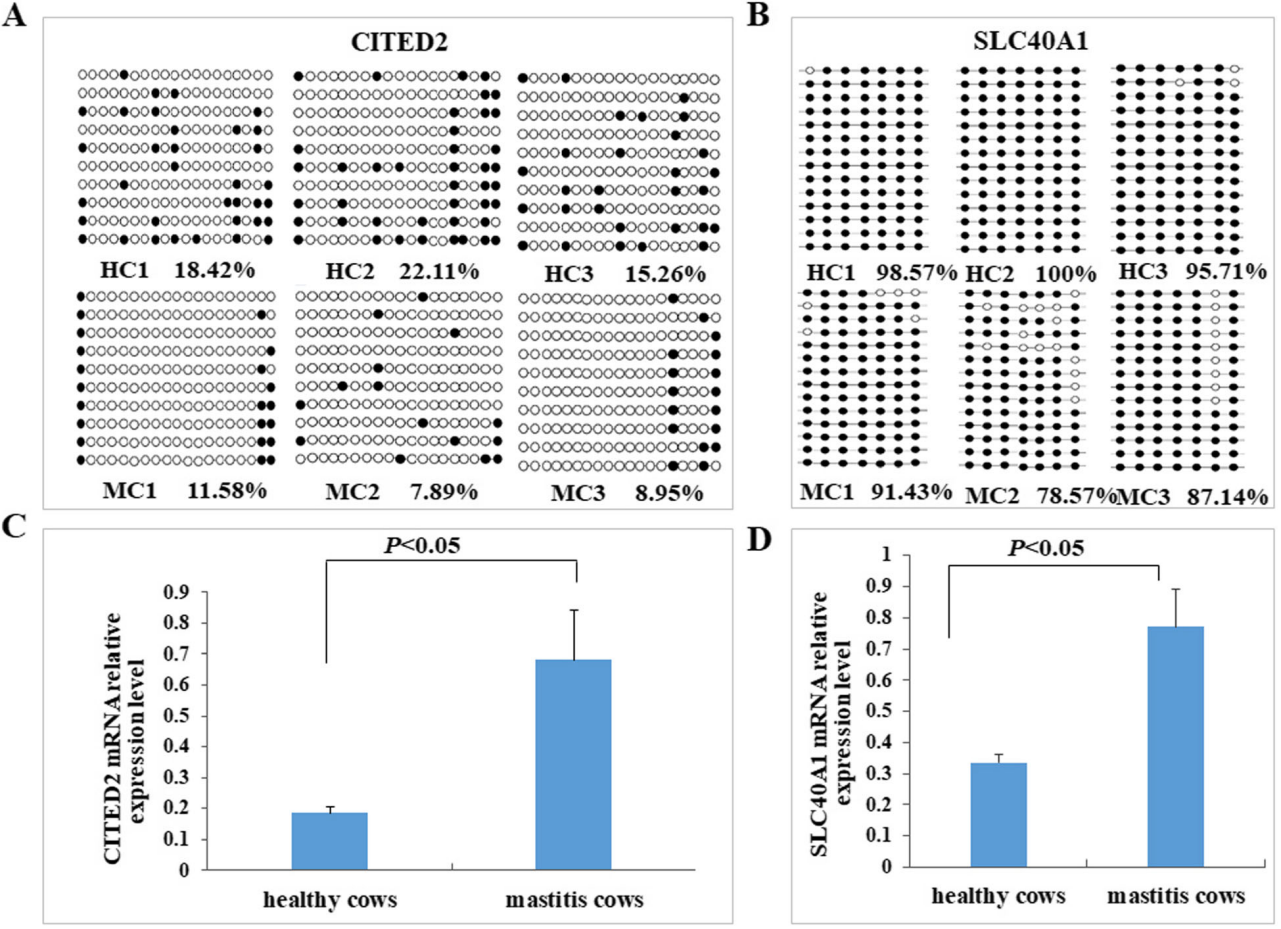
The methylation and expression levels of the *CITED2* and *SLC40A1* genes in the neutrophils of healthy and mastitic cows. Methylation level of *CITED2* gene (**a**) and *SLC40A1* gene (**b**) promoter region. Relative mRNA expression level of the *CITED2* gene (**c**) and the *SLC40A1* gene (**d**). Note: In (**a** and **b**), each row represents one clone, and each column represents one CG site. The open and solid circle indicate unmethylated and methylated CpGs, respectively

To further confirm the reliability of the transcriptomic and methylomic results by BSP and qPCR validation, the *CITED2* and *SLC40A1* genes with low methylation in promoter regions and high expression level were selected as candidate genes for analysis. The BSP sequencing results demonstrate that the average methylation levels of the promoter region of the CITED2 gene in healthy and mastitic cows were 18.60 and 9.47%, respectively (Fig. 6a). The difference was significant, and the methylation rate in the healthy group was higher compared with the mastitis group (*P* < 0.05). The average methylation levels of the promoter region of the *SLC40A1* gene in healthy and mastitic cows were 98.09 and 85.71%, respectively, which were significantly different (*P* < 0.05, Fig. 6b). The bisulfite sequencing results were almost consistent with the RRBS sequencing results. The relative expressions of *CITED2* and *SLC40A1* in healthy and mastitis cow blood neutrophils were detected using real-time PCR. The expression levels of the *CITED2* and *SLC40A1* genes in mastitis cows were significantly higher in healthy cows (*P* < 0.05) (Fig. 6c, d). Methylation and expression level validation experiments demonstrated that the *CITED2* and *SLC40A1* expression levels increased, and methylation levels decreased after *E. coli* infection. These results are in agreement with the results of high-throughput sequencing.

The *LGR4* gene methylation and expression levels analysis.

**Fig. 7:**
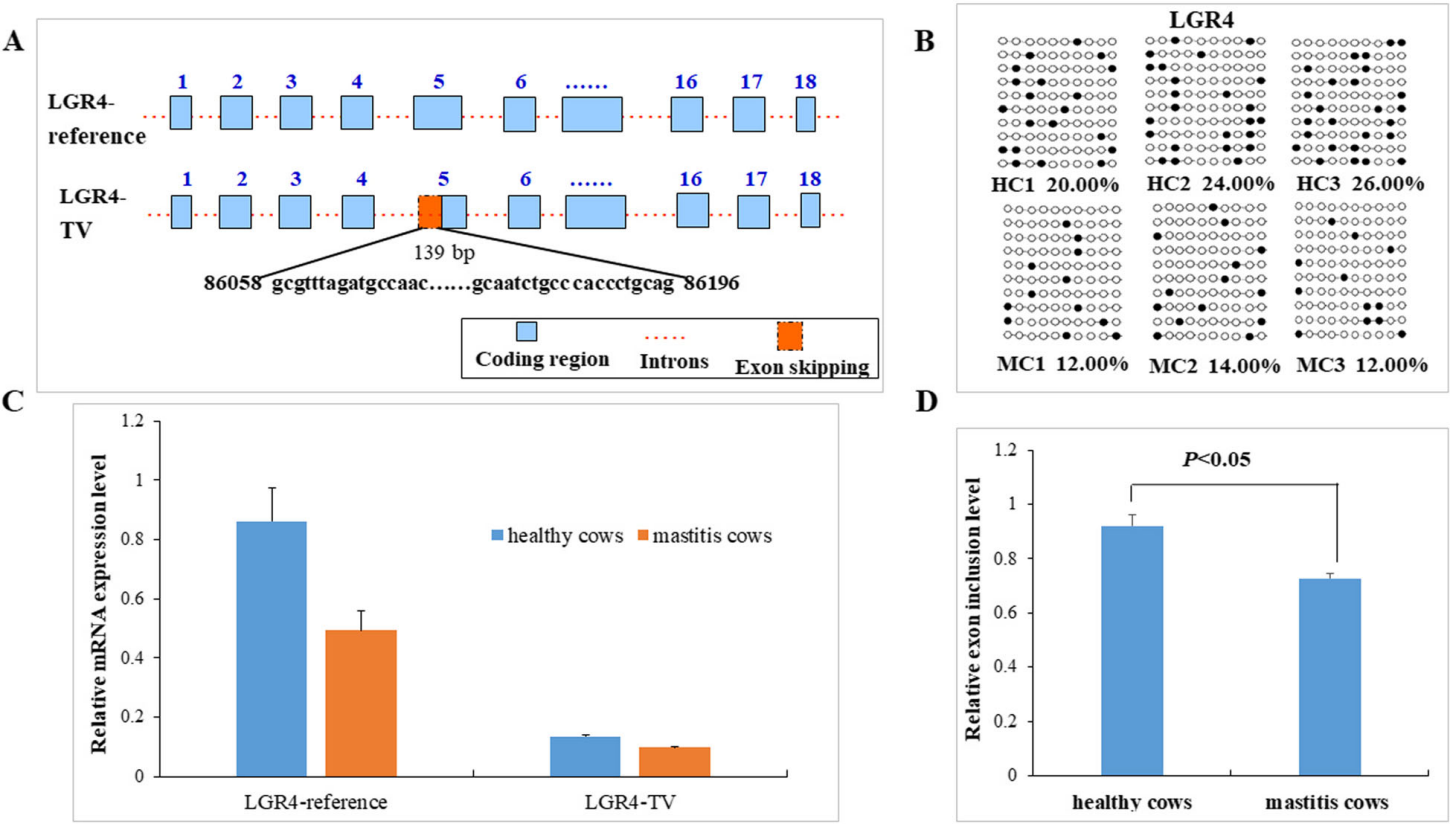
Experimental verification of the *LGR4* gene in the neutrophil of healthy and mastitic cows. **a** The schematic diagram of bovine LGR4-reference transcripts and alternative splicing transcripts LGR4-TV. **b** Methylation levels of the *LGR4* gene exon 5 region. **c** Relative mRNA expression levels of LGR4-reference and LGR4-TV transcripts. **d** The relative exon inclusion levels of the *LGR4* gene exon 5 Note: In (**d**), the relative exon inclusion level was calculated by dividing the amount of the inclusion isoform with the amount of the skipping isoform

The differentially methylated region of the *LGR4* gene was located on gene exon 5 in the RRBS sequencing. The *LGR4* gene exhibited alternative splicing pattern and differential expression in healthy and mastitic cows in the RNA-seq. DNA methylation plays an important role in exon recognition and alternative splicing regulation, which affected the splicing of pre-mRNA by influencing the recognition of alternative splicing exons [18, 19]. We amplified the differentially methylated region of the exon5 CpG island in the *LGR4* gene, and the amplified fragment contained 10 CpG sites. The PCR amplified fragment was ligated and transformed with the T3 vector, and positive clones were selected. Sixty clones with cytosine conversion rates above 95% (10 clones per cow) were selected for methylation analysis from healthy and mastitis samples. The average methylation level of the *LGR4* gene exon 5 in the healthy groups was 23.33%, and the average methylation level in the mastitic groups was 12.67%. The *LGR4* methylation level was decreased in the mastitic groups compared with the healthy groups (*P* < 0.05) (Fig. 7b).

To identify the presence of alternative splicing phenomenon in the exon 5 region of the *LGR4* gene, we amplified the coding region of the *LGR4* gene, and sequenced, and compared with the mRNA sequence of the *LGR4* gene. The alignment results showed that a new transcript, named LGR4-TV, was identified in the blood neutrophils of cows. The *LGR4* gene reference transcript was designated as LGR4-reference. The new transcript LGR4-TV missed part of exon 5 sequences with the length of 139 bp, which was the alternative 3′ splice sites pattern. Therefore, exon 5 of the *LGR4* gene is considered the alternative splicing exon (Fig. 7a). Real-time PCR results revealed that the LGR4-reference and LGR4-TV transcripts were expressed in blood neutrophils of healthy and mastitis cows. The expression levels of two transcripts LGR4-reference and LGR4-TV in healthy cows were significantly higher than mastitic cows (*P* < 0.05) (Fig. 7c). The LGR4-reference transcript displayed higher expression levels when compared with the LGR4-TV transcript in both healthy and mastitic cows (*P* < 0.05). We calculated the relative exon inclusion levels according to the amount of inclusion isoform and skipping isoform. The relative exon inclusion levels of the *LGR4* gene exon 5 were significantly higher in the neutrophil of healthy cows than in those of mastitic cows (*P* < 0.05) (Fig. 7d). The results indicated that the higher methylation level of the LGR4 gene exon 5 in the healthy groups may promote the inclusion level of the LGR4 gene alternative splicing exon 5.

Bta-miR-15a methylation and target gene *CD163* expression levels analysis.

**Fig. 8:**
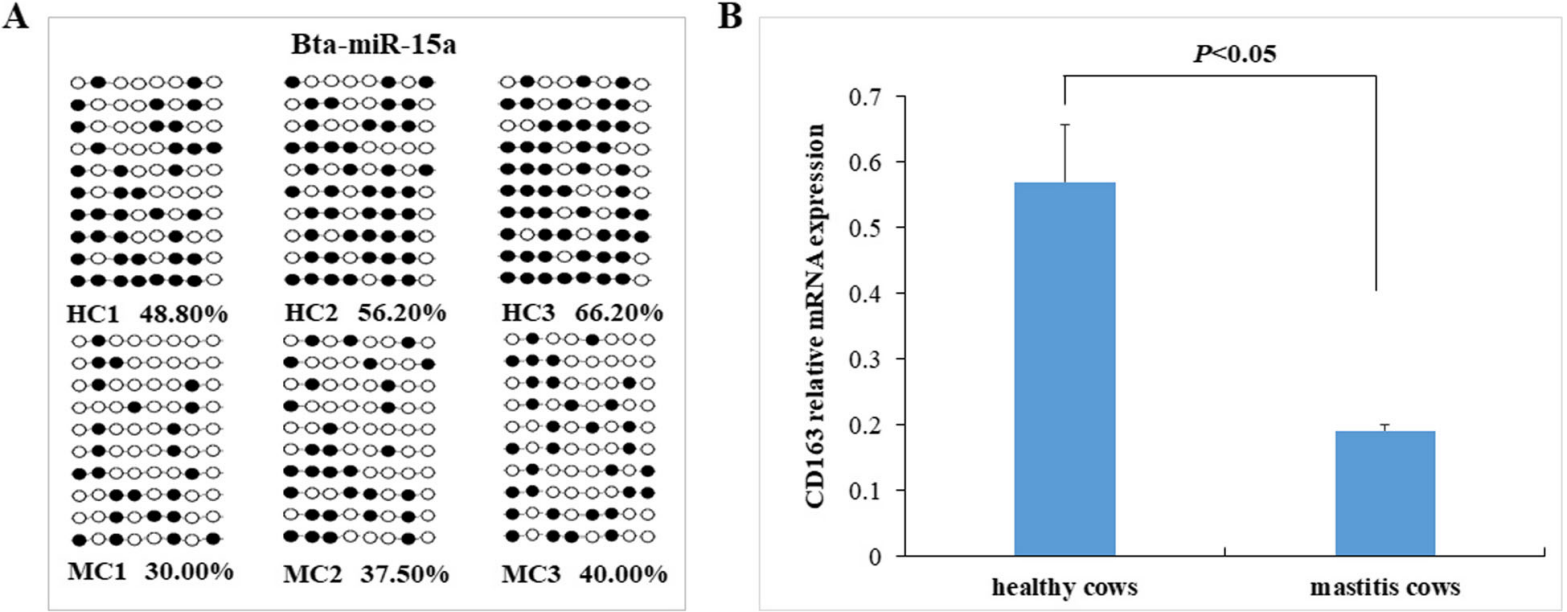
The bta-miR-15a methylation levels (**a**) and its target gene *CD163* expression levels (**b**) in neutrophils of healthy and mastitis cows

The BSP sequencing results showed that the average methylation level of bta-miR-15a promoter in healthy and mastitic cows were 57.06 and 35.83%, respectively (Fig. 8a). The methylation level of bta-miR-15a in the healthy group was significantly higher than in the mastitis group (*P* < 0.05). In our previous studies, the *CD163* gene was one of bta-miR-15a target genes [16]. The mRNA expression of the *CD163* gene in blood neutrophils were detected using real-time PCR. The expression levels of the *CD163* gene in healthy cows were significantly higher than mastitic cows (*P* < 0.05) (Fig. 8b). A positive correlation existed between the promoter methylation level of bta-miR-15a and the expression level of the target gene CD163.

## Discussion

This is the first report on DNA methylation and gene expression patterns of blood neutrophils in healthy and naturally *E. coli*-infected mastitic cows using RRBS and RNA-seq sequencing. We determined that overall DNA methylation levels of genome were decreased after *E. coli* infection. We demonstrated that DNA methylation in promoter regions affected the differential expression of immune-related genes, and DNA methylation in the exon regions regulated the alternative splicing pattern of genes. We also discovered key genes that play essential role in *E. coli* mastitis infection and identified genes *CITED2*, *SLC40A1,* and *LGR4* that regulate the differential expression of genes by DNA methylation in the *E. coli*-infected mastitic cow.

Cow mastitis is a prevalent disease that currently restricts dairy production. The incidence of mammary gland infection and clinical mastitis is usually highest during calving and early lactation. *E. coli* infection is the most common cause of mastitis during this period. During mastitis, leukocytes, such as neutrophils, migrate into the mammary gland. Shortly after mammary gland infection, the proportion of macrophages in somatic cells drastically decreases, whereas the proportion of neutrophils in somatic cells increases [20]. The accumulation of neutrophils at the site of infection is an important component of the inflammatory response and represents a hallmark of mastitis onset. Bovine neutrophils constitute the first intramammary defense against pathogen invasion. In the early stage of pathogen infection, sufficient number of neutrophils, which are rapidly recruited into the mammary gland, increase phagocytosis and prevent establishment of mastitis. In the present study, we extracted neutrophils from the milk venous blood and determined that the total amount of neutrophils in mastitic cows were higher compared with healthy cows.

With the development of epigenetics, DNA methylation plays a critical regulatory role in immune and inflammatory processes. DNA methylation is involved in disease susceptibility, including bovine mastitis. Song et al. [11] used MeDIP-chip assay to obtain the overall DNA methylation profiles of the bovine peripheral blood lymphocytes from healthy and *S. aureus* mastitic cows. The DNA methylation levels between the two groups indicated some differences in chromosome 11. The genome DNA methylation maps of healthy and *E. coli* mastitic cows across chromosomes were drawn in the present study. The DNA methylation levels in the neutrophil were down-regulated in *E. coli* mastitic cows compared with the healthy cows. The differences between the two research may be due to the differences in the experiment; lymphocytes were used in the study on *S. aureus*-infected mastitis cows, whereas this work used the neutrophil of *E. coli*-infected mastitic cows. Different pathogens can cause different immune responses in the mammary gland, which trigger different signaling pathways, thereby causing differences in the gene expression regulation of the corresponding pathways [21, 22].

The methylation levels of various genomic features differed. These methylation modifications are among the intrinsic mechanisms used to distinguish exons and introns during splicing. In this study, we analyzed the methylation levels in different genomic regions of promoters, exons, and introns. The methylation level of promoter sites were higher and rapidly decreased at the transcription start site. Consistent with previous studies, the methylation level of exons is higher than the methylation level of flanking introns. The methylation profiles, which involved strong DNA methylation at gene promoters, were associated with transcriptional silencing [23]. The function of a higher exons methylation level within the gene body may not prevent transcription but instead stimulate it [24, 25]. One possible role of DNA methylation within the gene body is to prevent pseudo-transcriptional activation by hidden internal promoters [26].

By GO and KEGG analysis, 52 differentially methylated genes were involved in immune, inflammatory, and defense responses; some of these play important roles in the course of neutrophils’ recognition of bacteria and moving toward the infection site. For instance, CD38, as the ADP-ribosyl cyclase enzyme, mediates neutrophils chemotaxis movement to the infected tissue by eliciting a sustained Ca^2+^ signal in cells [27]. CD38-deficient neutrophils are defective in chemotaxis and cannot accumulate in the infected tissue of mice [28]. Neutrophil cytosolic factor 4 (NCF4), as the component of phagocyte NADPH oxidase, is critical for host defense against microbial pathogens [29].

DNA methylation has a significant effect on miRNA expression, and approximately 10% of miRNA expression is regulated by DNA methylation [30]. miRNA also plays an important regulatory role in the immune and inflammatory responses of cows with mastitis [31, 32]. In the present study, we examined the differentially methylated miRNAs in healthy and *E. coli*-infected mastitic cows. Of these 26 differentially methylated miRNAs, bta-mir-15a and bta-miR-146a plays an important role in the regulation of mammary inflammation [16, 33]. In our study, the methylation level of bta-miR-15a was positively correlated with the expression level of its target gene *CD163*, thereby suggesting that DNA methylation of miRNA may regulate the expression of its target gene.

An association analysis of DNA methylation and gene expression data in the cow blood neutrophils was performed. As expected, a negative correlation between promoter methylation and gene expression levels was determined in this study. Twenty-nine differentially-expressed and -methylated genes were found in both RNA-seq and RRBS data. The *CITED2*, *SLC40A1*, and *LGR4* genes were included in the 29 differentially methylated and expressed genes. Lou et al. [34] reports that lipopolysaccharide (the *E. coli* bacterial cell wall components) induced *CITED2* expression via NF-kB in macrophages and negative feedback regulation of NF-kB signaling. The *SLC40A1* gene is expressed in various cell types, such as macrophages, and plays a role in innate immune response by chelation of iron [35]. The *LGR4* gene is a negative regulator of TLR2/4-related immune responses and plays an important role in innate immune responses by targeting *CD14* expression [36]. These results and literature suggest that the *CITED2*, *SLC40A1*, and *LGR4* genes can be used as candidate genes for *E. coli* mastitis resistance studies.

The *NRG1*, *MST1*, and *NAT9* genes are closely related with the progression of *S. aureus* subclinical mastitis and serve as epigenetic markers for improving mastitis resistance in dairy cows [11]. DNA methylation regulated the expression of different genes in cow mastitis caused by different types of bacteria. The possible reason is that different types of pathogens enter the cow’s mammary gland, secrete corresponding cytokines through different signaling pathways, and regulate differential gene expression.

In addition to the well-known effects of promoter methylation on the silencing of gene expression, DNA methylation is also involved in the regulation of gene alternative splicing [25]. Intragenic DNA methylation regulates alternative splicing exon inclusion levels by affecting the elongation rate of Pol II, or recruiting splicing factors [18, 19]. Zhang et al. (2018) reported that the exon 2 region of the *IL6R* gene had a higher level of DNA methylation in mastitic cow’s mammary gland tissues than healthy cows, thereby promoting the inclusion level of the *IL6R* gene alternative splicing exon 2 [10]. In the present study, a novel splice variant of the *LGR4* gene was characterized by the deletion of a 139 bp sequence in exon 5. The relative exon inclusion levels, and methylation levels of the *LGR4* gene exon 5 were significantly higher in healthy cows than in mastitic cows. Moreover, the healthy cow’s neutrophils displayed higher *LGR4* mRNA expression than the mastitic cow’s neutrophils. Therefore, we speculated that DNA methylation of the *LGR4* gene exon 5 may regulate the alternative splicing of the *LGR4* gene, thereby affecting its expression.

## Conclusions

This study investigated the function of DNA methylation in affecting transcription of protein-coding genes and miRNAs in cow mastitis caused by *E. coli*. Our data also suggested that DNA methylation affects the aberrant alternative splicing pattern of immune-related genes. Such studies greatly accelerate the progress in understanding the roles of DNA methylation in the mastitis infection process and providing new target genes and epigenetic markers for mastitis resistance breeding in dairy cattle.

## Methods

### Experimental animals and samples

Fifty-two age-matched Chinese Holstein candidate cows were selected from Jinan Jiabao Dairy Co. Ltd. in Jinan, Shandong, China. These animals were progenies of a validated Chinese Holstein bull. These cows range in age from 5 years old to 6 years old, including the third to fourth parity. All cows were fed total mixed ration (TMR) diets. Three healthy cows and three *E. coli*-infected mastitis half-sib cows from 52 candidate cows were finally selected for this study based on milk somatic cell count (SCC) records, hematological analysis, and bacteria identification results (Additional file 2: Figure S6). The blood and milk sample collections were permitted by the cattle owners. These experimental cows were not slaughtered after the procedure. The mastitis cows were treated with antibiotics and continued to be used for production. The healthy cows also continued to be used for production. The blood samples were taken from the milk vein of these cows and collected into sterile anticoagulant-containing vacuum tubes. The milk samples for SCC tests were collected thrice a day, with 8 h intervals, and mixed into preservative-filled tubes at a ratio of 4:3:3. The collection of milk samples for bacterial identification required the first three handfuls of milk to be discarded before collection and then collected into sterile tubes.

The mastitic cows were diagnosed according to clinical inflammation signs on the udder, such as redness, swelling, heat, and pain. The blood and milk samples of these mastitic cows were collected at the early stages of bacterial infection. The milk samples from infected lactating quarters of mastitic cows were collected for SCC tests and bacterial identification. The mastitic cows with milk SCC of over 1,000,000 per mL were selected. The selection criteria for healthy cow were as follows: normal breast development, negative bacteria culture results, and SCCs< 200,000/mL. Milk SCCs of cows were obtained by the Dairy Herd Improvement Laboratory, Dairy Cattle Research Center, Academy of Agricultural Sciences of Shandong Province, using a milk composition analyzer (Foss MilkScan FT 6000, Denmark). Bacteriological examinations were performed using Gram staining and 16S ribosomal DNA (16S rDNA) sequencing blast of bacterial genome. First, bacteria were isolated and cultured from bovine milk. Bacteria morphology was observed through Gram staining. Then, the bacterial 16S rDNA gene was amplified using a pair of universal bacterial primers [37]. The PCR products were sequenced and 16S rDNA homology analysis was conducted.

Simultaneously, 50 mL of peripheral blood samples containing EDTA anticoagulant were obtained from the milk vein of each healthy and mastitic cow for hematological analysis and separation of neutrophils. The blood constituent analysis was measured by animal blood analysis instrument. Peripheral blood neutrophils were isolated using the bovine peripheral blood neutrophil isolation kit (TBD Science, Tianjin, China) according to the manufacturer’s instructions. Quantification of neutrophils was performed as described in our previous publication [38].

### Reduced representation bisulfite sequencing and differential methylated regions analyses

Six DNA libraries were constructed, namely, three for healthy cows (HC1, HC2, and HC3) and three for mastitic cows (MC1, MC2, and MC3). Genomic DNA was fragmented using AIR DNA fragmentation kit (Bio Scientific Corporation), followed by end-repair and addition of 3′ A overhangs. Methylated sequencing adapters with 3′ T overhangs were ligated to the A tailed DNA fragments, and the ligation products were purified. The CpG-rich DNA fragments (40–220 bp) are selected by size, subject to bisulfite conversion. The bisulfite conversion of fragments were performed using EZ DNA methylation gold kit (Zymo Research, USA) according to the manufacturer’s protocol. The libraries were analyzed using Agilent 2100 Bioanalyzer (Agilent Technologies), quantified by real-time PCR, and sequenced on an Illumina HiSeq2500 sequencer at LC-BIO (Hangzhou, China). Sequencing was paired-end, and the read length was 100 bp. Quality control of the data was conducted using the FastQC (v0.11.4) software. Through the BSgenome software package, the valid reads were mapped according to the bovine reference genome (Btau3.1.1), and methylation calls were extracted.

The methylation levels were determined by dividing the reads covering each mC by the total number of reads. The differential methylated regions (DMRs) between healthy and *E. coli-*infected mastitis cow blood neutrophils were detected using MethylKit software package and edmr software [39]. In our analysis, the threshold for differentially methylated regions was set at *P* < 0.05.

The GO and KEGG enrichment pathway analyses were used to identify significant biological functions and pathways of differentially methylated genes. Protein interaction network analysis of differentially methylated genes were performed by STRING online software (https://string-db.org/cgi/input.pl). The differentially methylated genes located in the quantitative trait locus (QTL) associated with clinical mastitis (CM), somatic cell count, and somatic cell score (SCS) were screened through AnimalQTL (http://www.animalgenome.org/cgi-bin/QTLdb/BT/search). The differentially methylated miRNAs between HCs and MCs libraries were also detected.

### Transcriptome sequencing and differentially expressed genes analyses

Total RNA were extracted and purified from bovine peripheral blood neutrophils of HC and MC group samples. Subsequently, the six cDNA libraries were constructed and sequenced using PE technology (2 × 100 bp read length) on the Illumina Hiseq 2500 machine at LC-BIO (Hangzhou, China). The construction protocols of cDNA library and sequencing were conducted as previously described [40]. The raw data were processed by removing low quality reads (containing sequencing adaptors reads, sequencing primer reads, and nucleotide with a q quality score lower than 20) to obtain valid data. Valid data were mapped to the bovine reference genome (Btau3.1.1). The differential expression levels of genes were calculated using FPKM method between the HC and MC groups. The expressed genes with an absolute value of log2 ratio of ≥1 and *P* < 0.05 were defined as differentially expressed genes (DEGs).

### Correlation analysis between methylation and transcription data

Pearson correlation coefficient between DNA methylation and gene expression data of healthy and mastitic cows were calculated using SPSS statistics 17.0. The differentially-methylated and -expressed genes were identified in both RNA-seq and RRBS sequencing data. The potential target genes of differentially methylated miRNAs were predicted by TargetScan software (http://www.targetscan.org/vert_72/).

### Bisulfite PCR sequencing

To confirm the reliability of RRBS data, the methylation level of three immune and inflammation-related genes *CITED2*, *SLC40A1*, and *LGR4* were identified using bisulfite sequencing polymerase chain reaction (BSP). The procedure of BSP was conducted according to our previous method [41]. Briefly, genomic DNA of the peripheral blood neutrophils from HC and MC group samples was modified using BisulFlash DNA modification kit (Epigentek, USA). The modified DNA was used in PCR amplification. Two pairs of primer for each gene were designed using Methyl Primer Express Software v1.0 for nested PCR amplification. The primer sequences used for nested PCR are listed in Additional file 1: Table S6. The PCR products were purified, cloned into pEASY-T3 vectors, and transformed into DH5ɑ cells. Ten positive clones for each gene per sample were randomly selected for sequencing. The sequence results were processed through BiQ Analyzer software.

### Quantitative real-time PCR

The expression levels of the *CITED2* and *SLC40A1* genes were identified by quantitative real-time PCR (qPCR). The cDNA of peripheral blood neutrophils from HC and MC group samples was amplified using SYBR® Premix Ex Taq™ II (TaKaRa, Dalian, China). The primers of target genes amplification are shown in Additional file 1: Table S6. The qPCR was monitored using a Roche LightCycler 480 machine (Roche Applied Science, Mannheim, Germany). The bovine *β-actin* gene was used as the housekeeping internal control gene [42]. Relative quantification of target gene expressions was done using the 2^−△△Ct^ method.

### Alternative splicing identification, mRNA expression and relative exon inclusion level analyses of the *LGR4* gene

A pair of primers of the LGR4-cDNA (Additional file 1: Table S6) were designed to amplify the coding region of the *LGR4* gene based on the LGR4 mRNA reference sequence (GenBank, NM_001205511). The PCR products were electrophoresed, purified, ligated to the pEASY-T3 vector, and transformed into competent cell Trans5α. Positive clones were selected and sent to the company for direct sequencing. The sequencing results were aligned with bovine LGR4 mRNA sequence using the DNAStar software.

A new transcript LGR4-TV was identified in the blood neutrophils of cow. The *LGR4* gene reference transcript LGR4-reference and splice transcript LGR4-TV were subjected to quantitative expression analysis in blood neutrophils from HC and MC group samples using qPCR. The relative exon inclusion levels of the *LGR4* gene in neutrophils of healthy and mastitis cows were calculated by dividing the amount of inclusion isoform with the amount of the skipping isoform.

### Bta-miR-15a methylation and target gene CD163 expression levels analysis

The methylation level of bta-miR-15a was analyzed by bisulfite sequencing PCR. In previous studies, our results indicated that CD163 gene was the target gene of bta-miR-15a by luciferase activity assay [16]. Therefore, the expression levels of the CD163 gene was also identified by quantitative real-time PCR. The procedure of bisulfite sequencing PCR and quantitative real-time PCR were as described above. The primers of bta-miR-15a nested PCR and CD163 gene quantitative amplification are listed in Additional file 1: Table S6.

## Supplementary information

**Additional file 1: Table S1.** Summary of the differentially methylated genes in the two libraries. A total of 494 DMRs including 61 up-methylated genes and 433 down-methylated genes were identified in the healthy and mastitic groups. **Table S2.** Summary of the differentially methylated genes harboring in the QTLs associated with clinical mastitis, somatic cell count and somatic. **Table S3.** Summary of the differentially methylated miRNAs in the two libraries. A total of 26 differentially methylated miRNAs including 8 up-methylated miRNAs and 18 down-methylated miRNAs were identified in the healthy and mastitic groups. **Table S4.** Summary of the differentially expressed genes in the two groups. A total of 1339 DEGs including 1094 up-regulated genes and 245 down-regulated genes were identified in the healthy and mastitic groups. **Table S5.** Summary of differentially methylated and expressed genes in the RRBS and RNA-seq sequencing. **Table S6.** The primers used for BSP and qPCR.

**Additional file 2: Figure S1.** KEGG enrichment analysis of differentially methylated genes (A) and GO enrichment analysis of differentially expressed genes (B) in healthy and *E. coli* mastitic cows neutrophils. **Figure S2.** Protein interaction network analysis of differentially methylated genes (A) and -expressed genes (B) in healthy and *E. coli* mastitic cows’ neutrophils. **Figure S3.** The correlation analysis of gene promoter methylation level and gene expression level in MC and HC groups. **Figure S4.** KEGG enrichment analysis of genes with differential methylation and expression. **Figure S5.** Regulatory network of differentially methylated miRNAs (yellow circles) in the RRBS sequencing and their putative target genes (blue circles) in the RNA-seq. **Figure S6.** The selection of half-sibling healthy and *E. coli* mastitic cows.

## Data Availability

The RRBS and RNA-seq clean data from this study were deposited in NCBI Sequence Read Archive with accession numbers PRJNA533860 and PRJNA534172, respectively (https://submit.ncbi.nlm.nih.gov/subs/sra/).

## Abbreviations

16S rDNA: 16S ribosomal DNA
BSP: Bisulfite sequencing PCR
CM: Clinical mastitis
DEGs: Differentially expressed genes
DMRs: Differential methylated regions
*E. coli*: *Escherichia coli*
GO: Gene ontology
HCs: Healthy cows
KEGG: Kyoto encyclopedia of Genes and Genomes
MCs: Mastitic cows
microRNAs: miRNAs
qPCR: Quantitative real-time PCR
QTL: Quantitative trait locus
RNA-seq: Transcriptome sequencing
RRBS: Reduced representation bisulfite sequencing
SCC: Somatic cell count
SCS: Somatic cell score
TMR: Total mixed ration
