# Supplementary material for: Genome-wide methylation and transcriptome of blood neutrophils reveal the roles of DNA methylation in affecting transcription of protein-coding genes and miRNAs in E. coli-infected mastitis cows
Source: BMC Genomics. 2020 Jan 30;21:102. doi: 10.1186/s12864-020-6526-z (PMC6993440; doi:10.1186/s12864-020-6526-z)
Supplement: Supplementary file 2 — Additional file 2: Figure S1. KEGG enrichment analysis of differentially methylated genes (A) and GO enrichment analysis of differentially expressed genes (B) in healthy and E. coli mastitic cows neutrophils. Figure S2. Protein interaction network analysis of differentially methylated genes (A) and -expressed genes (B) in healthy and E. coli mastitic cows’ neutrophils. Figure S3. The correlation analysis of gene promoter methylation level and gene expression level in MC and HC groups. Figure S4. KEGG enrichment analysis of genes with differential methylation and expression. Figure S5. Regulatory network of differentially methylated miRNAs (yellow circles) in the RRBS sequencing and their putative target genes (blue circles) in the RNA-seq. Figure S6. The selection of half-sibling healthy and E. coli mastitic cows. [file 12864_2020_6526_MOESM2_ESM.docx]

**
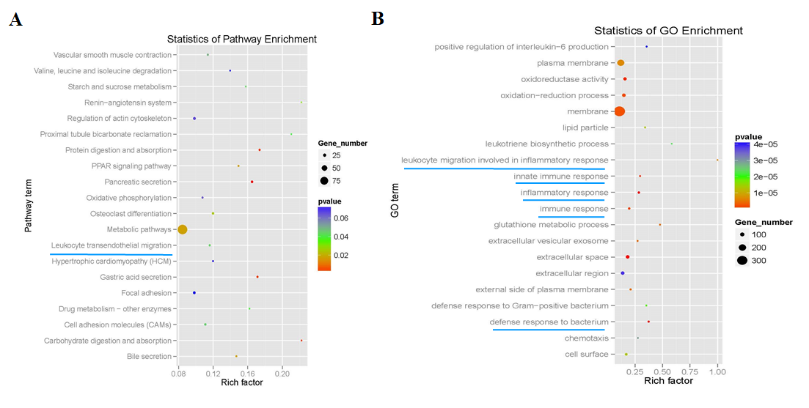
**

**Figure S1.** KEGG enrichment analysis of differentially methylated genes (A) and GO enrichment analysis of differentially expressed genes (B) in healthy and *E. coli* mastitic cows neutrophils.

**
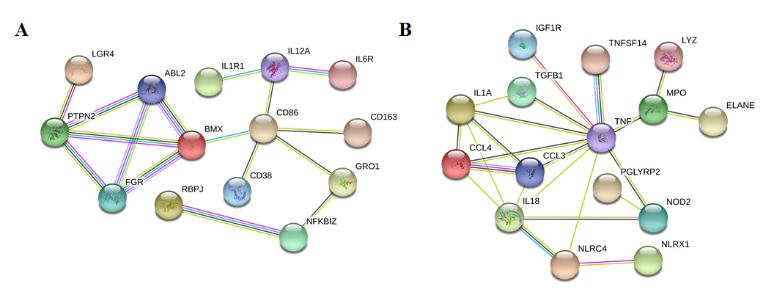
**

**Figure S2.** Protein interaction network analysis of differentially methylated genes (A) and -expressed genes (B) in healthy and *E. coli* mastitic cows’ neutrophils.

**
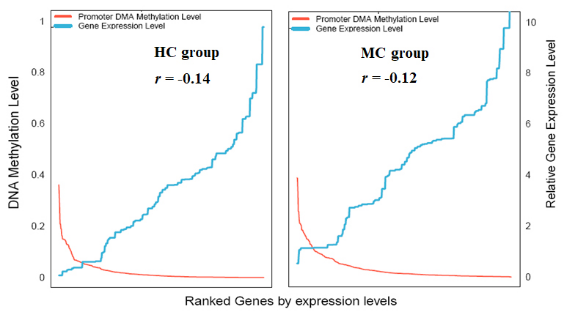
**

**Figure S3.** The correlation analysis of gene promoter methylation level and gene expression level in MC and HC groups.

**
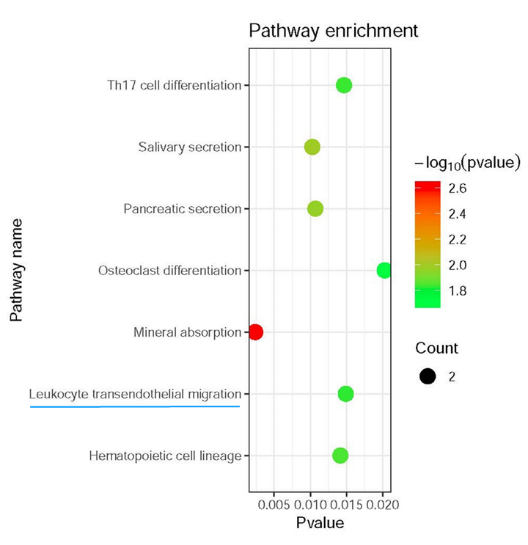
**

**Figure S4.** KEGG enrichment analysis of genes with differential methylation and expression.

**
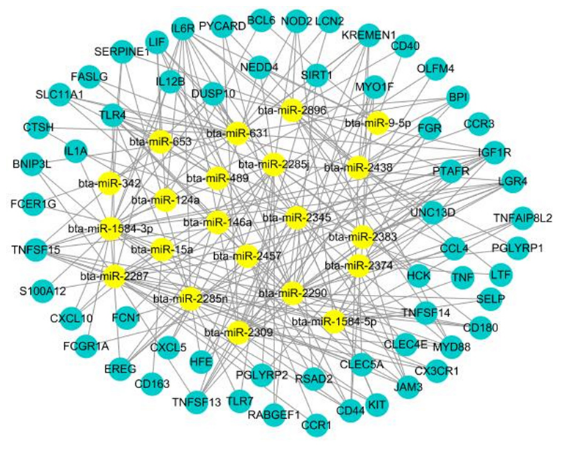
**

**Figure** **S5.** Regulatory network of differentially methylated miRNAs (yellow circles) in the RRBS sequencing and their putative target genes (blue circles) in the RNA-seq.

**
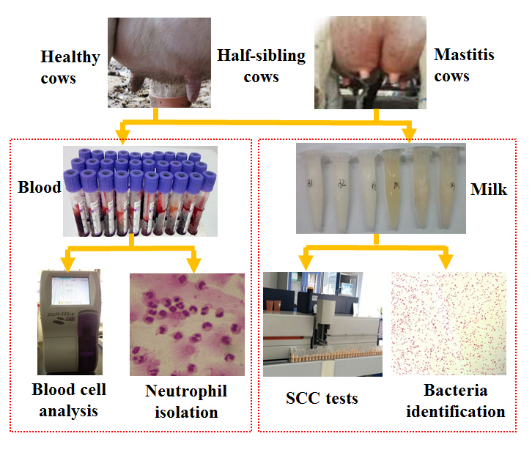
**

**Figure S6.** The selection of half-sibling healthy and *E. coli* mastitic cows.
